# Identification of Candidate Auxin Response Factors Involved in Pomegranate Seed Coat Development

**DOI:** 10.3389/fpls.2020.536530

**Published:** 2020-09-15

**Authors:** Li’ang Yu, Chunyan Liu, Jiyu Li, Botao Jia, Xiaoxiao Qi, Ray Ming, Gaihua Qin

**Affiliations:** ^1^ Key Laboratory of Horticultural Crop Genetic Improvement and Eco-physiology of Anhui Province, Institute of Horticulture Research, Anhui Academy of Agricultural Sciences, Hefei, China; ^2^ Department of Plant Biology, University of Illinois at Urbana-Champaign, Urbana, IL, United States; ^3^ Key Laboratory of Fruit Quality and Developmental Biology, Anhui Academy of Agricultural Sciences, Hefei, China; ^4^ Center for Genomics and Biotechnology, Fujian Provincial Key Laboratory of Haixia Applied Plant Systems Biology, Fujian Agriculture and Forestry University, Fuzhou, China

**Keywords:** auxin response factor, gene expression, gene family evolution, phylogenetics, seed coat development

## Abstract

Auxin response factors (ARFs) are transcription factors, regulating the auxin signaling pathways involved in plant development and related processes. In this study, we performed the genome-wide identification and characterization of *ARF*s in pomegranate and compared them with *ARF*s from three other species. Seventeen PgrARFs were identified and clustered into four groups, according to their phylogenetic relationship with the remaining 59 ARFs. A recent whole-genome duplication event in pomegranate may have contributed to the expansion and diversification of *PgrARF*s. Genomic truncation and variant splicing mechanisms contributed to the divergence of *PgrARF*s, a conclusion that was supported by different exon-intron structures of genes and incomplete conserved domains of PgrARFs in a specific phylogenetic group (group III). Interestingly, the absence of motifs from certain *PgrARF* genes corresponded to their low transcription levels, which contrasted to the highly expressed *PgrARF*s with intact motifs. Specifically, *PgrARF1* and *PgrARF2* highly expressed in both inner and outer seed coat, and phylogenetically related to *Arabidopsis* orthologs which mediates cell divisions in seed coat. We infer these two *PgrARFs* might involve in seed coat development through cell divisions in response to auxin regulation. These findings provided information on the characteristics and evolutionary relationships of *PgrARF*s, but also shed lights on their potential roles during seed coat development in pomegranate.

## Introduction

Pomegranate (*Punica granatum* L.) (2n = 2x = 18) belongs to the family Lythraceae, and is widely cultivated in countries with Mediterranean-like climates around the world, including Tunisia, Turkey, Spain, Egypt, Morocco, the USA, China, India, Argentina, Israel, and South Africa (Qin et al., 2017). The pomegranate is widely consumed in the form of fruits, juice, wines, and medicines due to its nutritional, medicinal, and ornamental values (Kim et al., 2002). In addition, pomegranate differs from other fruit trees in terms of its unique seed structure, with a compressed inner seed coat and an expanded fleshy outer seed coat (Qin et al., 2020). The expanded fleshy outer seed coat is the major edible part that largely determines the yield and edible rate. Thus, genetic studies of seed coat development could benefit pomegranate improvement and production.

The development of seed coat is regulated and orchestrated by several transcription factors (TFs) such as ARF, MADS-box, and WRKY (Nesi et al., 2002; Garcia et al., 2005; Schruff et al., 2006; Fang et al., 2019). Among those TFs, *AtARF2* from auxin response factor (ARF) family was identified involving in cell divisions of seed coat, supported by a *mega integument* (*mnt*) mutant allele of *AtARF2*, which induce extra cell divisions and organ growth in seed coat (Schruff et al., 2006). Particularly, cell divisions of seed coat in early seed development stage accompanies with the proliferation of endosperm. The process further constrains the cavity of embryo development in later stages and limit the seed size and content (Sun et al., 2010). Moreover, auxin mediates *ARF* expression and activates seed coat development by removing the function of a Polycomb Group (PcG) protein-encoded gene, which epigenetically blocks seed coat development (Figueiredo et al., 2016). Therefore, exploration of the ARFs regulations and auxin signaling pathways provides valuable evidence to understand the genetic mechanism of seed coat development.

ARFs activators and auxin/indole acetic acid (Aux/IAA) repressors are two TFs co-regulate auxin signaling pathway. ARFs target the auxin-response genes by binding to promoters of auxin response DNA elements (AuxREs), which contain the TGTCTC element, to suppress or activate the transcription level of auxin response-related genes. ARFs contain three major domains, namely a conserved N-terminal B3-type DNA-binding domain (DBD), a variable middle region (MR), which acts as an activation or suppression region for ARFs, and a C-terminal dimerization domain (CTD) for protein dimerization (Piya et al., 2014). Meanwhile, ARFs are mediated by Aux/IAAs in an auxin concentration-dependent manner. Low auxin concentrations induce the formation of Aux/IAA protein heterodimers, which inhibit ARF activity and repress ARF transcription, whereas higher auxin concentrations derepress ARF activity through degradation of Aux/IAAs from the SCF ^TIR1/AFB^ pathway (Guilfoyle and Hagen, 2012; Wang and Estelle, 2014). Understanding the regulatory mechanisms of ARFs is key to understanding the auxin signaling pathways.

ARFs have been widely identified in plants along with multiple copies in each species. Twenty-three ARFs have been identified in *Arabidopsis* (*Arabidopsis thaliana*), 25 in rice (*Oryza sativa*), 21 in tomato (*Solanum lycopersicum*), and 19 in sweet orange (*Citrus × sinensis*) (Okushima et al., 2005; Wang et al., 2007; Xing et al., 2011; Li et al., 2015). Nearly doubled numbers of ARFs were identified from banana (*Musa acuminata*), soybean (*Glycine max*), and rapeseed (*Brassica napus*), which is explained by whole-genome duplications (WGD) or polyploidizations (Hu et al., 2015; Singh and Jain, 2015; Wen et al., 2019). Also, conserved domains exhibited truncation or amino acid substitution in some species. For example, 14, eight, 11, and seven truncations of the DBD domain were identified from barrel medic, maize, sweet orange, and tomato (Zouine et al., 2014; Shen et al., 2015). The roles of a few ARFs involved in seeds, leaves, flowers, and fruits development have been characterized by functional validations. In *Arabidopsis*, *AtARF7* and *AtARF19* control leaf expansion and lateral root growth (Okushima et al., 2005; Wilmoth et al., 2005), and *AtARF5* and *AtARF8* are critical elements related to flower formation and fruit development (Hardtke and Berleth, 1998; Goetz et al., 2006; Liu et al., 2018). In tomato, *SlARF3* was characterized as a strong candidate for the differentiation of epidermal cells and trichomes (Zhang et al., 2015), whereas *SlARF9* participated in the regulation of cell division during the process of fruit development (De Jong et al., 2015). However, the role of ARFs in seed coat development has been rarely investigated.

In this study, we attempted to explore potential relations between ARFs and seed coat development in pomegranate. Integrated analyses of phylogenetic classification, exon-intron structure, domain structures, of ARFs from pomegranate were conducted and compared with three other species, includes those ARFs from *Arabidopsis*, grape, and eucalyptus. We compared ARFs copy number variation to grape, a species with a recent genome triplication (Jiao et al., 2012). Also, we identified colinear *PgrARFs* in eucalyptus, a species as the same family of pomegranate, to explore potential ARF lineage-specific diversification. Further, *PgrARFs* with intact structure and high expression level in seed coat were chosen and carefully studied, including their temporal expression in different growth stages, correlation between expression and seed coat content increment, and their relations to functional orthologs from *Arabidopsis*. Our study could provide fundamental information about *PgrARFs* characteristics, evolution, and structural variation, also, the candidate ARFs we chose could provide references to study seed coat development in pomegranate.

## Materials and Methods

### Identification of ARFs and Reconstruction of a Phylogenetic Tree

Protein sequences of pomegranate (*P. granatum*), *Arabidopsis* (*A. thaliana*), eucalyptus (*Eucalyptus grandis*), and grape (*Vitis vinifera*), with genome annotations, were downloaded from Phytozome (v11.1, https://genome.jgi.doe.gov) for local sequence blast. Primary genome-wide identification of ARFs from the four species was performed using the hidden Markov model (HMM) by HMMER (Johnson et al., 2010) as described in protocols. Briefly, the domain profiles of B3 (PF06057) and Auxin_resp (PF02362) from the Pfam database (https://pfam.xfam.org/) were searched for, using the “HMMsearch” function with a threshold E-value <1e-05 against the local database. Furthermore, sequences identified above were confirmed by searching for their conserved domains from the Pfam database and from SMART (http://smart.embl-heidel-berg.de), with sequences with incomplete annotation information or missing domains being removed manually. To construct the phylogenetic tree, full-length protein sequences of ARFs selected according to the above criteria were aligned using the multiple sequence alignment tool (MUSCLE) (Edgar, 2004). Conserved domains, namely the DBD, MR, and CTD domains, were identified from amino acid sequences, based on alignment positions derived from previous studies (Bailey et al., 2009). Before the construction of the phylogenetic tree, an optimum amino-acid substitution selection model was selected using model-generator tools (Keane et al., 2006). The phylogenetic tree was constructed using the PhyML tool, based on the maximum-likelihood (ML) method (starting tree: BIONJ; bootstrap:100; tree topology search: NNIs). The final tree was visualized using the interactive tree of life (iTOL) (Letunic and Bork, 2006; Rambaut, 2007). Based on the classification of the phylogenetic tree, variations in amino acid sequences in each ARF group were further analyzed by conducting pairwise alignments through BLASTp.

### *ARF* Gene Structure Analysis and Identification of Conserved Motifs of ARFs

The gene structure of each *ARF*, including exon-intron distribution, was displayed, based on published gene annotation information from the four species, using the Gene Structure Display Server (GSDS) (Hu et al., 2014). In addition, the number of genes, number of exons, average gene length and average exon length of ARFs in each group in each species were determined. The conserved motifs for each ARF protein sequence were identified by MEME (http://meme-suite.org/tools/meme), with eight as the maximum motif number for comparison. The sequence for each conserved motif identified by the MEME search was confirmed, based on the hits classification by BLAST against the conserved domain database (https://www.ncbi.nlm.nih.gov/Structure/cdd/), the location of each type of motif being represented by different symbol shapes and colors from iTOL. The final conserved motif pattern for each ARF was organized, based on groups and orders from the phylogenetic tree.

### Chromosomal Localization and Synteny Analysis of *PgrARFs*

To investigate the gene evolution of *ARF*s from pomegranate and eucalyptus, another closely related species from the Lythraceae family, comprehensive gene synteny and duplication analyses were conducted between eucalyptus and pomegranate using MCScanX (Wang et al., 2012) and visualized by Circos (Krzywinski et al., 2009). Initially, the genomic location of 17 ARFs from eucalyptus and 17 ARFs from pomegranate were mapped to their respective chromosomal locations, based on annotation from the Phytozome database (https://phytozome.jgi.doe.gov/pz/portal.html) (v11.1). Furthermore, the protein sequences of the 34 ARFs and any ARFs within the 100-kb flanking regions were retrieved for protein sequence alignment. The pairwise alignment for each sequence was conducted by BLASTp, with an E-value >1e-05 and an identity score >35% as cut-offs. Synteny analysis was conducted by MCScanX (http://chibba.pgml.uga.edu/mcscan2/) with the following settings: match-score: 50; overlap-window: 5; E-value: 1e-05; max-gaps: 25. The non-synonymous mutation rate (dN), synonymous mutation rate (dS) and the ratio of non-synonymous to synonymous substitutions (dN/dS) were calculated for each collinear ARF pair identified from pomegranate. Also, duplication analysis from the two species was conducted by classifying duplication mode using the *duplicate_gene_classifier* function in MCScanX, with default settings based on protein sequences.

### Plant Material for *ARF* Time-Course Gene Expression Studies

Pomegranate cultivar “Dabenzi”, a major cultivar of pomegranate grown in Anhui Province in China, was selected to study the relationship between seed coat development and *ARF* gene expression, by sampling tissues at several time points during fruit maturation. “Dabenzi” trees were planted in an orchard in Anhui Province in China (Huaiyuan, 32°95’N, 117°19’E), and the flowers in full bloom were labelled and classified as 0 days after full bloom (DAFB) in spring 2019. We sampled nine fruits at each time point from a 30-year-old “Dabenzi” tree, namely 25, 60, 90, 116, and 145 DAFB. Each fruit sample was dissected manually and 100 seeds from each fruit were randomly selected for weighing, with three biological replicates (with three fruits randomly selected to represent each replicate). For gene expression analysis, the outer seed coats from three sets of the replication collected from 25, 66, 90, 116, and 145DAFB were manually squeezed and frozen in liquid nitrogen, then stored at -80°C prior to RNA extraction. Isolation of RNA was conducted using an OmniPlant RNA Kit (DNase I) (CwBiotech, Taizhou, China) and cDNA synthesis was carried out by EasyScript One-Step gDNA Removal and cDNA Synthesis SuperMix (Transgen, Beijing, China), following the protocols described by the manufacturers.

### Relative Expression of Candidate *PgrARF* Genes During Seed Coat Development

To identify the candidate *PgrARF*s potentially involved in seed coat development, we collected the global transcriptomic data of pomegranate from published data, which derived from several plant tissues, namely root, flower, leaf (each organ sample collected at one stage of fruit development), peel (three stages), inner seed coat (three stages), and outer seed coats (each organ samples collected at 50, 95, and 140 days after pollination (DAP) and labeled as Stage1, Stage2, and Stage3) for screening (Qin et al., 2017). Only those *PgrARF*s that exhibited high expression levels in both inner and outer seed coats were selected for further qPCR analysis. The primers used in qPCR for these selected *ARF*s were designed by Primer Premier 5.0 software (http://www.premierbiosoft.com), following BLAST against the reference genome of pomegranate to prevent amplification of non-specific products. To prevent false positives from qPCR, the cDNA samples from 5-time points, with three technical replications and three biological replications at each time point, were used, and qPCR was performed using the LightCycler 96 SYBR GREEN I Master (Roche, Indianapolis, IN, USA) in a 20-µl reaction volume, according to the manufacturer’s protocol. The relative expression levels calculated by the cycle threshold (Ct) 2(^-ΔΔCt^) method, with a pomegranate actin gene (OWM91407) as an internal control (Zhao et al., 2015). Results from different samples were compared, using the two-tailed t-test (α = 0.05). Further, increment of 100-seed weight and differences in relative transcriptional levels of candidate *ARF*s between any two representative growth stages were also compared by Pearson correlation analysis. Any *PgrARF* with a strong correlation may putatively involve in pomegranate seed coat development.

### Potential Divergence of Duplicated *PgrARF*s

Combining the identification of duplicated genes with transcriptomics data was used to address the problem of potential effects of gene duplication on gene function divergence. In this study, we compared the correlation of gene expression from different tissues among duplicated *PgrARF* gene pairs as determined by Pearson’s correlation coefficient. We proposed the use of significant correlation coefficient values to verify the degree of expression difference: r < 0.3 signified divergence, 0.3 < r < 0.5 signified ongoing divergence, and r > 0.5 signified non-divergence, based on previous studies (Blanc and Wolfe, 2004).

## Results

### Identification and Phylogeny of ARFs

**Figure 1:**
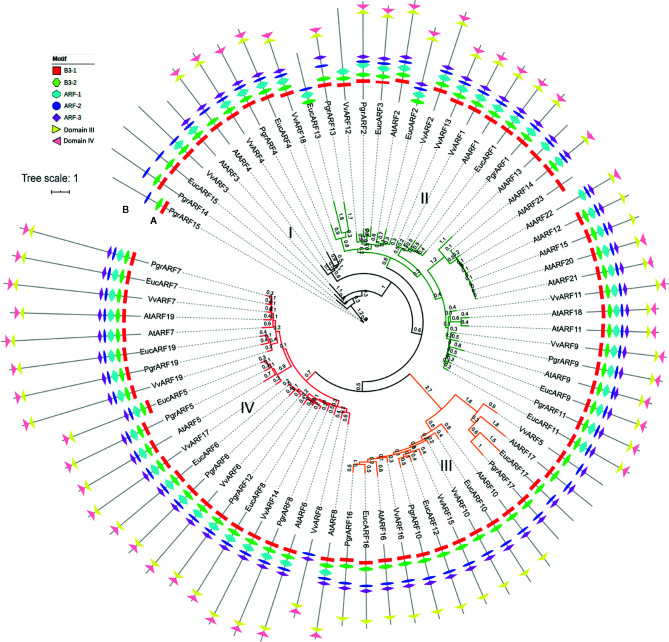
Phylogenetic tree and motif structure of ARFs from four plant species. **(A)** The phylogenetic tree was reconstructed with the maximum likelihood (ML) method in PhyML and represented in a circular fashion. The four groups are marked in four different colors, namely black, green, orange and red for groups I, II, III, and IV, respectively. Branch length was marked on each branch of the tree. **(B)** Seven motifs corresponded to each ARF, identified as B3, ARF activation/depression or IAA/Aux motifs, are represented by different symbols as shown in the legends.

A total of 76 ARFs were identified from the four species studied, 17 in pomegranate (*P. granatum*), 23 in *Arabidopsis* (*A. thaliana*), 17 in eucalyptus (*E. grandis*), and 19 in grape (*V. vinifera*) (**Supplementary S1**). Based on the sequence alignment feature, the Jones-Taylor-Thornton (JTT) amino-acid substitution model proved to be the optimum model for further phylogenetic tree reconstruction. According to the phylogenetic tree, the 76 protein sequences were clustered into four groups (**Figure 1A**). All ARFs were renamed, based on their potential orthologs from *Arabidopsis*, or sequentially, if no corresponding ortholog was found (**Supplementary S2**). The 76 ARFs were distributed unevenly among the four groups, as group I contained the smallest number of ARFs (nine), whereas group II consisted of the largest number (31). Interestingly, more than half of the ARFs from *Arabidopsis* were clustered in group II whereas the ARFs from pomegranate were distributed more evenly among the four groups, similar to the situation with grape and eucalyptus ARFs (**Supplementary S3**).

**Figure 2:**
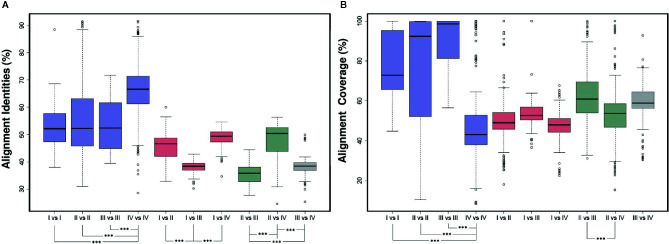
Pairwise alignment comparison of ARF protein sequences. **(A)** Comparison of pairwise sequence identity of full-length ARF proteins was conducted in ten groups, namely I vs. I, II vs. II, III vs. III, IV vs. IV, I vs. II, I vs. III, I vs. IV, II vs. III, II vs. IV, and III vs. IV. The boxplot shows the median, interquartile range, and maximum and minimum scores of each data set. Outliers are shown as black circles beyond the whiskers. The level of significance was marked in asterisk. **(B)** Comparison of pairwise sequence coverage of full-length ARF proteins was conducted in ten groups, namely I vs. I, II vs. II, III vs. III, IV vs. IV, I vs. II, I vs. III, I vs. IV, II vs. III, II vs. IV, and III vs. IV. The boxplot shows the median, interquartile range, and maximum and minimum scores of each data set. Outliers are shown as black circles beyond the whiskers. The level of significance was marked in asterisk.

The pairwise alignment between the two ARF protein sequences in each pair of the 76 ARFs revealed some noteworthy features (**Figure 2A**). Within the same group, we found that ARFs from group IV shared a significant higher average identities (P < 2.2e -16), with those from group I, group II, and group III exhibiting lower but similar average identities (P > 0.05). Further, we observed a significantly lower identities from comparison between two different groups which contains sequences from group III, including I vs III, II vs III, and III vs IV (P < 1.8e -16). We also noticed that ARFs from group II exhibited the highest degree of deviation from those from groups I, III, and IV, indicating a higher level of diversity of the ARF protein sequences in group II. Additionally, we found some contrasting features of alignment coverage, compared with identities (**Figure 2B**). Interestingly, genes from group IV exhibited the highest identities but the lowest coverage values, which significantly differed with I vs I, II vs II, and III vs III (P < 1.4e – 15). Comparison of II vs III exhibiting the lowest identities but the highest coverage values among all the two-group comparisons. These could be attributed to the variations in gene length and gene structure as a result of genomic deletions.

### Diversified ARF Protein Sequences Among the Four Groups

To identify the sequence features of *ARF*-encoded proteins, multiple alignments of the 76 ARF protein sequences were used to identify conserved amino acid residues and the distribution of conserved domains (**Supplementary S4**). Based on the alignments, DNA-binding domain (DBD) from the N-terminal region was identified as residues in between 190 and 310, the middle region of the ARF activation/repression domain at 370–520, and the C-terminal dimerization (CTD) domain at 1350–1450. Among the three domains of the ARF proteins, DBD was the most conserved regions, along with three conserved residues across the 76 sequences at K^271^, G^276^, and D^277^, with these three residues possibly being closely associated with key functions of ARFs.

To further elucidate the variation in motif patterns among the 76 protein sequences, we performed the motif analysis by searching for conserved motif distribution in each ARF amino acid sequence. The MEME search identified eight conserved motifs which could be classified as two specific B3 motifs (namely the B3–1 and B3–2 motifs) from the DBD domain, three ARF activation/suppression-related motifs (namely the ARF-1, ARF-2, and ARF-3 motifs), and two Auxin-related motifs from CTD domains (namely the domain III and domain IV motif). Overall, motif patterns diversified and correlated with the distribution of ARFs based on the phylogenetic tree (Fig. 1b). We found that all ARFs from group III lacked the ARF-1 motif and the domain IV motif. PrgARF17, EucARF17, AtARF14, and VvARF5 also lacked the domain IV motif, compared with the rest of the group III members. Interestingly, we observed a longer branch distribution for all group III ARF proteins from the phylogenetic tree, as well as ARFs bearing longer branches with the most severe motif loss. The different lengths of tree branches indicated the recent emergence of ARFs from duplications. We hypothesized that the more recent evolution of these *ARF* genes from group III could be associated with motif loss from the DBD domain and the CTD domain. Additionally, losses of the ARF-1, ARF-3, domain III, and domain IV motifs from PgrARF14, PgrARF15, and EucARF15 were observed, a situation which was more severe than that occurring with encoded proteins of potential orthologs from *Arabidopsis* and grape (*AtARF3, AtARF4*, and *VvARF3*). This might be explained by lineage-specific variation of *ARF* genes from pomegranate and eucalyptus, both members of the Lythraceae family. The other ARFs suffering motif loss were EucARF13, AtARF13, and AtARF13 in group II and VvARF3 and AtARF3 in group I, which were less closely related to the respective phylogenetic patterns.

### Gene Truncation and Gene Structure Variations of *ARFs*

**Figure 3:**
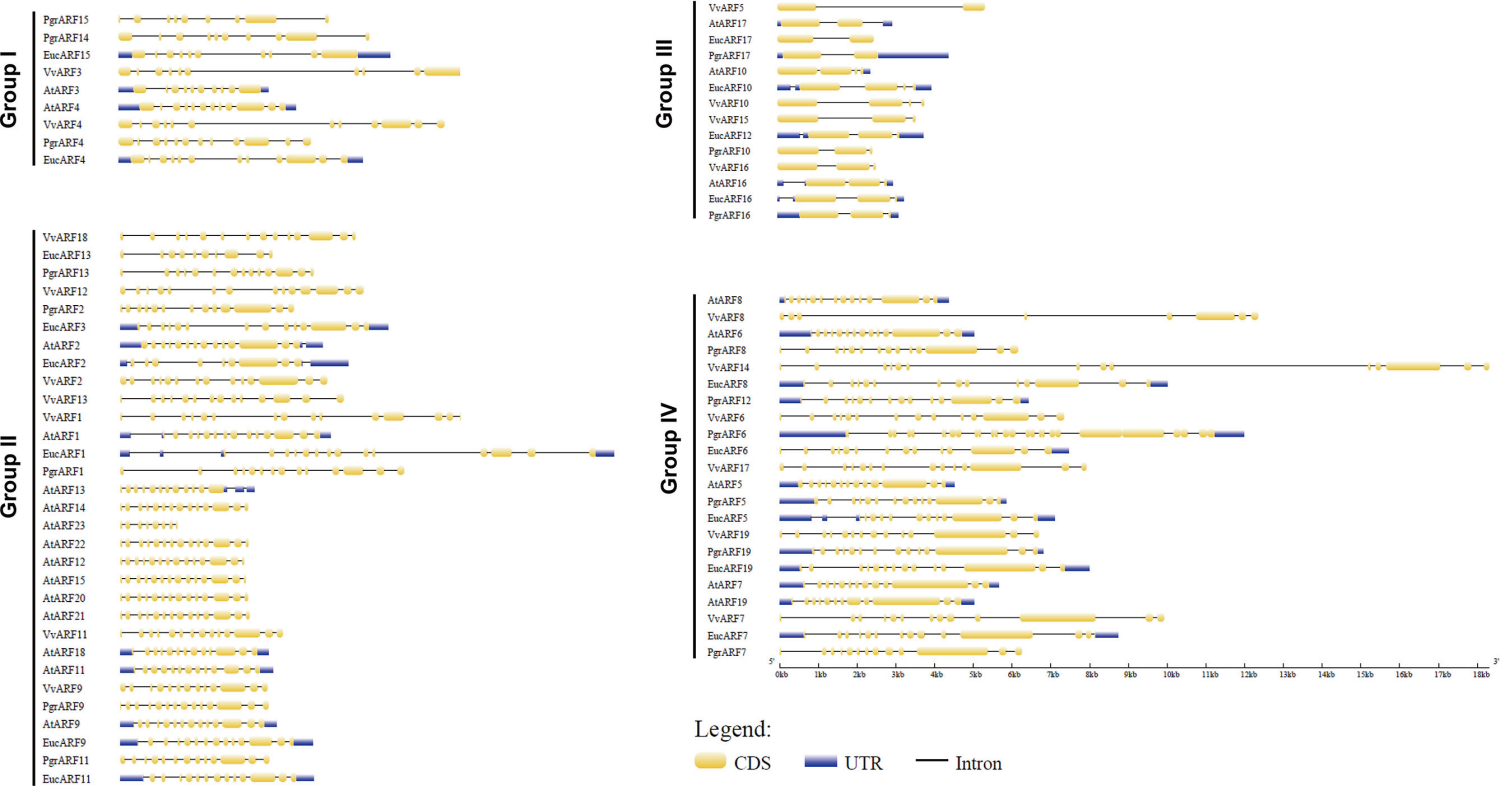
Exon–intron structure of 76 *ARF*s full-length genomic sequences. The intron/exon structure of *ARF*s was visualized by gene structure display server (GSDS). Yellow rectangles represent exons and dark lines represent introns, with UTRs (untranslated regions) being marked with shaded rectangles.

To explore the putative causes of protein sequence divergence, we compared the structural gene annotation from the four species. The complete gene annotation information, which included untranslated regions (UTRs), exon sites and intron sites, presented a diversified genomic pattern. Among the 76 genomic *ARF* sequences, we found that the genomic sequences of *ARF*s from eucalyptus were longer than those from the other three species, due mostly to their longer introns (**Figure 3**). Most *ARFs* from the same group contained similar numbers of exons among the four species but different exon numbers were identified among the four phylogenetic groups. Surprisingly, significantly shorter (25%–33% length) genes and substantially longer (2- to 3-times length) exons were observed in genes from group III, compared with the genes from the other three groups, with fewer exons on average per gene (**Supplementary S3**).

### Duplication and Evolution of *ARF*s From Eucalyptus and Pomegranate

**Figure 4:**
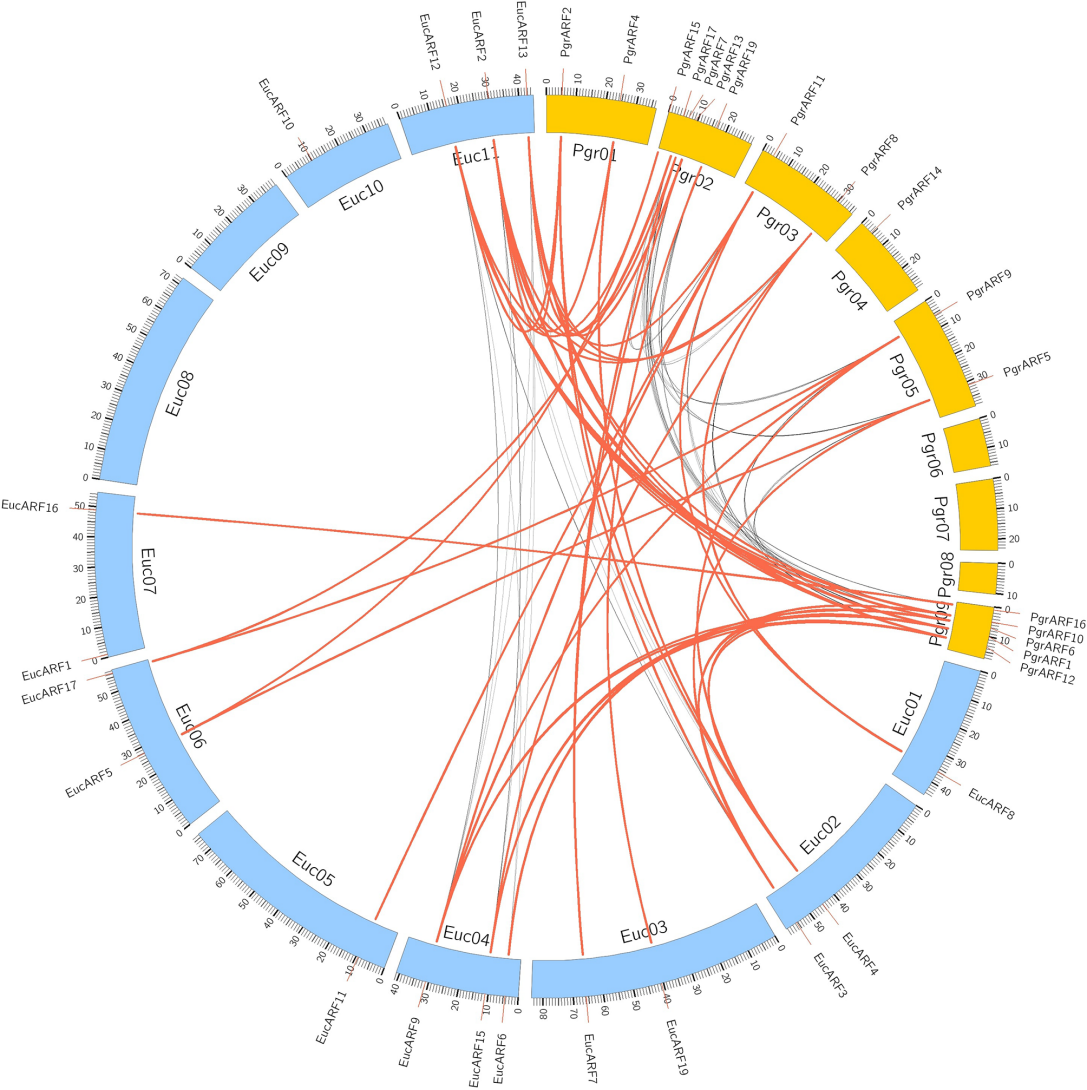
Collinearity of *ARF*s between eucalyptus and pomegranate. Genes from 100-kb flanking genomic regions of 17 *PgrARF*s and 17 *EucARF*s were mapped on chromosomes of pomegranate and eucalyptus, based on gene annotation. The red lines connect inter-specific collinear gene pairs, whereas black lines connect collinear genes within each species.

Identification of collinear gene pairs and homologous genes enabled the identification of duplicated gene pairs. In the present study, pairwise comparisons between *ARF*s and genes from each 100-kb flanking region from the pomegranate and eucalyptus genomes revealed 32 collinear blocks from 22 genomic location combinations (E-value: 1e-05). The 32 collinear blocks were classified between pomegranate and eucalyptus, with six blocks within the pomegranate genome, but only two blocks within the eucalyptus genome (**Table 1**). Collinear regions between pomegranate and eucalyptus were mainly distributed on two eucalyptus chromosomes and one pomegranate chromosome (Euc04, Euc11, and Pgr09), whereas collinear regions within the same species were distributed on Euc11 and Pgr09 (**Figure 4**). Analysis of duplication type revealed that whole-genome duplication (WGD) contributed to nearly all duplicated *ARFs* from pomegranate, similar to the finding in eucalyptus (**Supplementary S5**). Collinear gene-pair distribution and duplication types revealed that 12 collinear gene-pairs were categorized in group IV (**Table 2**). We speculated that *PgrARF*s from group IV played a substantial role in *ARF* expansion in pomegranate, caused by the recent WGD in this species.

**Table 1:** Summary of collinear *ARFs* from pomegranate.

Gene 1	Gene 2	Class	Gene expression	Correlation	dN	dS	dN/dS
*PgrARF11*	*PgrARF1*	II–II	non–divergent	0.714	0.47	2.1	0.22
*PgrARF9*	*PgrARF1*	II–II	non–divergent	0.512	0.51	2.52	0.2
*PgrARF17*	*PgrARF16*	III–III	non–divergent	0.561	0.67	1.36	0.49
*PgrARF19*	*PgrARF6*	IV–IV	divergent	0.207	0.54	1.66	0.32
*PgrARF19*	*PgrARF8*	IV–IV	divergent	0.436	0.53	2.57	0.2
*PgrARF7*	*PgrARF5*	IV–IV	divergent	0.602	0.55	2.25	0.25
*PgrARF7*	*PgrARF6*	IV–IV	divergent	-0.139	0.53	2.11	0.25
*PgrARF8*	*PgrARF12*	IV–IV	NA	NA	0.19	1.03	0.18
*PgrARF13*	*PgrARF10*	II–III	divergent	0.332	0.98	2.7	0.36
*PgrARF11*	*PgrARF12*	II–IV	NA	NA	0.68	1.67	0.41
*PgrARF9*	*PgrARF6*	II–IV	divergent	0.142	0.71	1.64	0.43
*PgrARF17*	*PgrARF9*	III–II	divergent	0.332	1.04	1.81	0.57
*PgrARF5*	*PgrARF1*	IV–II	divergent	0.351	0.76	1.48	0.51
*PgrARF7*	*PgrARF11*	IV–II	divergent	0.201	0.77	1.25	0.61
*PgrARF8*	*PgrARF1*	IV–II	divergent	0.429	0.75	1.21	0.62
*PgrARF5*	*PgrARF10*	IV–III	divergent	0.061	0.95	1.41	0.67
*PgrARF7*	*PgrARF16*	IV–III	divergent	0.063	1.13	1.1	1.02

**Table 2:** Summary of collinear genes between pomegranate and eucalyptus.

Classification	Location	Block no.	Collinear gene pairs
Pomegranate–ucalyptus	Euc02-Pgr01	2	24
	Euc02-Pgr05	1	7
	Euc02-Pgr09	1	6
	Euc03-Pgr02	1	8
	Euc04-Pgr03	1	11
	Euc04-Pgr05	1	6
	Euc04-Pgr09	2	12
	Euc05-Pgr03	1	11
	Euc06-Pgr02	1	10
	Euc06-Pgr05	1	9
	Euc07-Pgr09	1	11
	Euc11-Pgr01	1	6
	Euc11-Pgr02	1	9
	Euc11-Pgr03	1	6
	Euc11-Pgr09	4	38
Pomegranate–Pomegranate	Pgr02-Pgr03	1	7
	Pgr02-Pgr05	2	13
	Pgr02-Pgr09	2	14
	Pgr03-Pgr09	2	14
	Pgr05-Pgr09	2	12
Eucalyptus–Eucalyptus	Euc02-Euc11	1	8
	Euc04-Euc11	2	14
Total	22	32	256

### Expression Characteristics of *PgrARF*s in Different Tissues of Pomegranate

**Figure 5:**
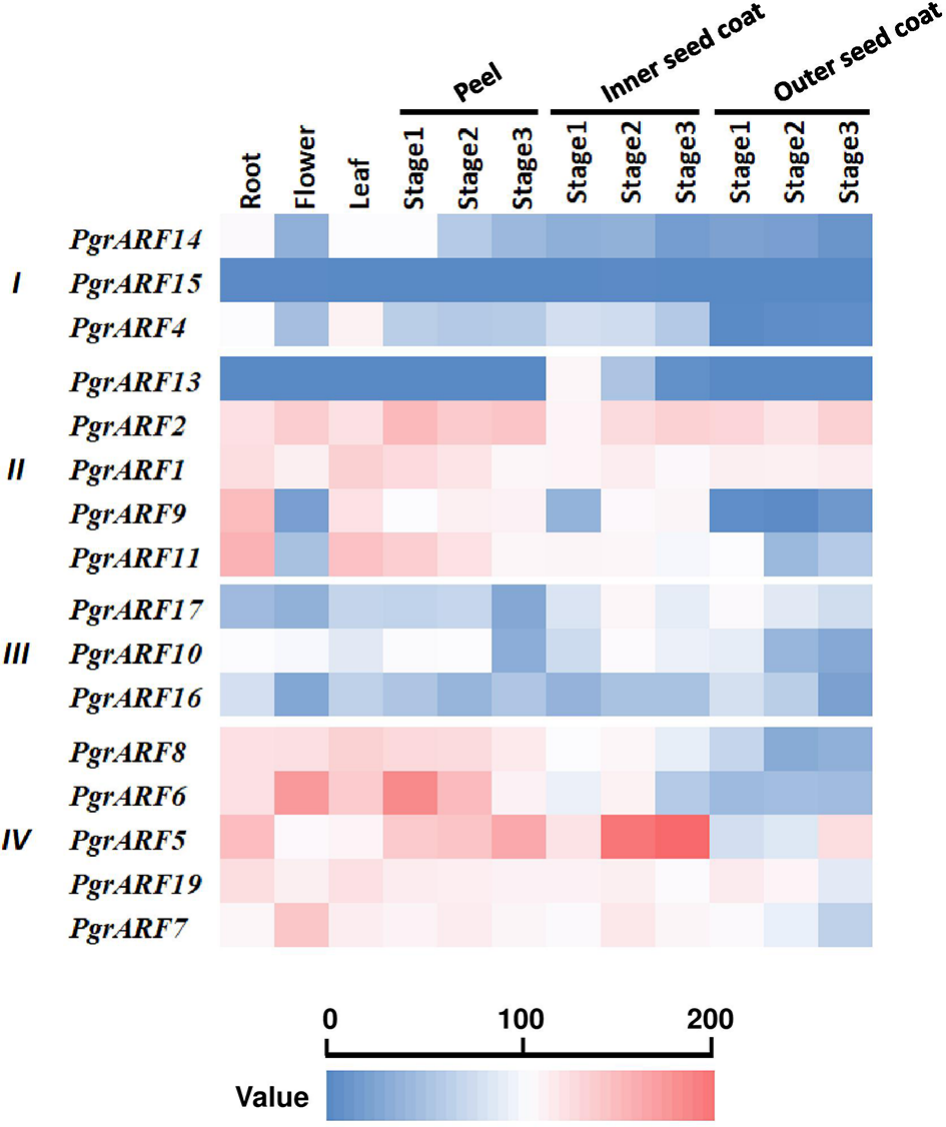
Transcriptome profiling of *PgrARF*s from different plant tissues. Relative expression level of 16 *PgrARF*s from root, flower, leaf (all sampled at one stage), peel (three stages), inner seed coat (three stages), and outer seed coat (three stages) of pomegranate are presented as Reads Per Kilobase of transcript per Million mapped reads (FPKM) values, based on phylogenetic grouping. Three stages for peel, inner seed coat, and outer seed coat were 50, 95, and 140 days after pollution (DAP).

Transcriptome profiling from a variety of plant tissues may help to identify tissue-specific genes which, in turn, helps to identify candidate genes for specific biological processes. In this study, tissue-specific transcriptomic data, including root, flower, leaf, peel, and seed coat (inner and outer seed coat), revealed variations in expression patterns among the four *PgrARFs* groups. We identified two highly expressed *ARFs* with a broad spectrum of expression during vegetative growth and reproductive developmental stages, namely *PgrARF1* and *PgrARF2* from group II (**Figure 5**). Expression of another three *ARFs* (*PgrARF5*, *PgrARF7*, and *PgrARF19*) was up-regulated in most tissues except for down-regulation in the outer seed coat. However, nearly all the genes from groups I and III were transcribed at extremely low levels. The remaining genes exhibited partially tissue-specific expression. For instance, *PgrARF9, PgrARF11, PgrARF6*, and *PgrARF8* were highly expressed in root, leaf, and peel. We also compared the transcriptome pattern of collinear genes to detect any differences in gene expression. Among 17 pairs of collinear genes from pomegranate, two pairs of collinear genes from group II and one pair from group III revealed non-divergent patterns of expression. However, expression of five gene pairs from group IV was less closely correlated, despite the similar structures shared over much of the genes. The nine gene pairs showing different expression patterns might be explained by structural variations among groups. In all, a majority of collinear *PgrARFs* were differentially expressed, potentially due to structural variation among different groups or some other unknown factors.

### Identification of *ARF* Candidates Involved in Seed Coat Development

On the basis of transcriptome profiling of *PgrARFs*, we selected *ARF* candidates that were potentially involved in seed coat development, and performed qPCR to confirm the results, then carried out statistical analysis of the relationship between 100-seed weight and mRNA abundance level. Transcriptome data revealed that *PgrARF1* and *PgrARF2* were highly expressed in both the inner and outer seed coats, suggesting that these patterns might be related to their involvement in seed coat development. *PgrARF5*, *PgrARF7*, and *PgrARF19*, which were highly expressed in the inner seed coat, were expressed at a lower rate in the outer seed coat during fruit development. It is suggested that these three genes might participate in the early stages of seed coat development while being less involved in the later stages.

**Figure 6:**
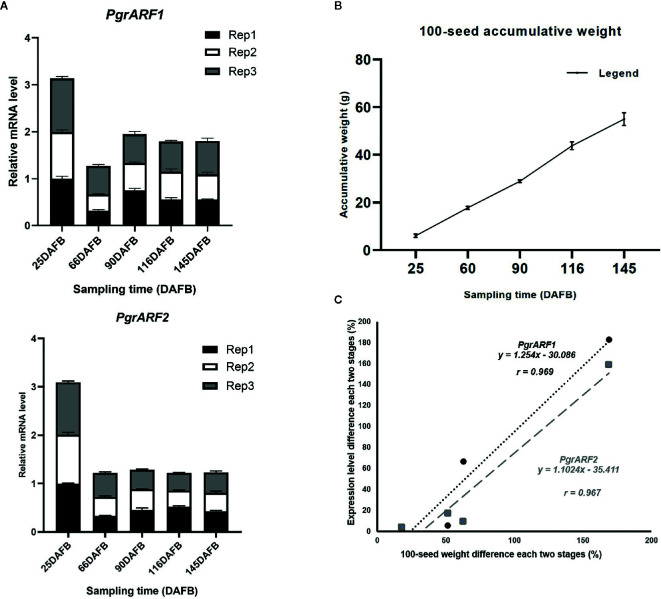
The relationship between *PgrARF* gene expression level and pomegranate seed weight. **(A)** Relative gene expression level of *PgrARF1* and *PgrARF2* genes for outer seed coats at 25, 55, 90, 116, 145 days after full bloom (DAFB) with three technical replicates and three biological replicates. **(B)** 100-seed pomegranate seed weight (g) from three biological replicates across five stages of fruit development (days after full bloom, DAFB). **(C)** Linear regression analysis of differences in 100-seed weight from each of two stages with respect to change in rate of gene expression from four sampling time-points.

Similar expression patterns of *PgrARF1* and *PgrARF2* were detected by transcriptomics and by qPCR. The expression of these two candidate *PgrARF*s peaked at 25 DAFB then fell to their lowest level at 55 DAFB, followed by a slight increase or decrease at the third time point (**Figure 6A**). Regression analysis revealed a significant linear relationship between 100-seed weight increment and mRNA transcription level differences in each two stage of each candidate gene, as identified by significant positive correlations for both *PgrARF1* (*r* = 0.969, P < 0.05) and *PgrARF2* (*r*= 0.967, P < 0.05) (**Figures 6B, C**). Hence, we propose that *PgrARF1* and *PgrARF2* might be involved in the outer seed coat development of pomegranate.

## Discussion

### Gene Duplication as Trigger of *PgrARF* Expansion

Gene duplication is one of the major driving forces for plant genome evolution, and also impacts the expansion of and functional variation within gene families. Global identification of *ARFs* from multiple flowering plant lineages has identified the co-occurrence of *ARF* expansion and whole-genome duplication (WGD) (Jiao et al., 2011; Hu et al., 2015; Le et al., 2016; Wen et al., 2019). It is plausible to propose gene duplication as a trigger of *PgrARF* expansion. In the present study, we identified that WGD contributed to *ARF* gene duplication in pomegranate, which mostly occurred in groups II, III, and IV, matching the distribution pattern from previous studies (Finet et al., 2013). Interestingly, duplicated (collinear) ARFs from group IV were functionally divergent, whereas three pairs of collinear genes within groups II and III exhibited correlated expression patterns (**Table 2**). Similar examples of functional redundancy of *arf6/arf8* mutants from *Arabidopsis* and duplicated *AtARF3* and *AtARF4* with functional divergence had been identified in previous studies (Nagpal et al., 2005; Finet et al., 2010). In all, we proposed that gene duplication is closely related to gene redundancy or functional divergence during the evolution of the *ARF* gene family in pomegranate.

### Genomic Truncation and Splicing Variation Contributed to Diversified *PgrARF*s

Modification at the post-transcriptional level is another major force potentially contributing to gene diversification. Alternative splicing of the *ARF* gene family has been identified from numerous land plants (Finet et al., 2013; Zouine et al., 2014). For instance, different functional roles were identified from two isoforms of *Arabidopsis* *ARF4 (ARF4* and δ*ARF4)* during carpel development (Finet et al., 2010). In another species in the Lythraceae family, alternative transcripts have been identified from 10 out of 17 *ARFs* from eucalyptus (Yu et al., 2014). On the other hand, no alternative transcripts were found among the 17 *PgrARF*s, based on an exhaustive search of putative transcripts from annotation, although eucalyptus shared quite a few collinear *ARFs* with pomegranate, and highly similar ARF protein sequences were identified from inter-specific pairwise alignment (**Table 1**). Consequently, the origins of alternative splicing of *ARFs* might be lineage specific, and hence, less relevant to the evolution of species. In addition, reduced exon numbers, increased gene lengths and truncated genomic lengths were identified from group III (**Supplementary S3**). It is plausible to reason that, besides the missing residues or motifs from ARF protein sequences, some functional divergence might have occurred due to these structural gene variations. However, this hypothesis, regarding the structural variants which, in pomegranate, appeared exclusively in group III, indicates that the variation in structure was not confined to pomegranate but was also found in the other three species in our study. We hypothesized that this specific phenomenon during the subfamily evolution might be related to the evolution of the splicing process. It would be tempting in future studies to explore the potential mechanisms involved in achieving increased exon length.

### Conserved Domains as Evidence of Intact *PgrARF* Function

Conserved amino acid residues or motifs play substantial roles in maintaining intact domain functions, which is closely related to gene expression and to gene regulation. The role of each domain from the ARF protein was characterized in numbers of earlier studies (Guilfoyle and Hagen, 2012; Korasick et al., 2014; Guilfoyle, 2015). The DBD achieves binding to the DNA target site in an auxin-independent manner. On the other hand, the MR in the ARF domain either activates (the Q-rich ARF domain) or represses (the S-rich ARF domain) transcription level, whereas the CTD regulates the auxin response pathway by interaction with Aux/IAAs. Numerous truncated proteins caused by motif losses were identified from several of the species under investigation, whereas such variations in domains showed a close relationship between gene expression and sequence conservation. For example, significantly reduced transcription levels in root, leaf, shoot, cotyledon and flower were exhibited by nine *MtARF* genes from *Medicago*, all of which exhibited the missing CTD or the partially truncated ARF domain (Shen et al., 2015). A similar expression pattern in citrus revealed a lower relative mRNA abundance from *CiARF3* and *CiARF17*, which could be related to the missing CTD domain (Li et al., 2015). In our structural analysis and expression profiling (**Figures 1B** and **5**), we found that ARFs with incomplete ARF and CTD domains, lacking ARF-1, domain III, and domain IV motifs from certain PgrARFs from both group I and group III, were associated with low transcription rates from a number of plant tissues.

In addition, several residues have been reported to play a substantial role in ARF transcription (Boer et al., 2014). In *Arabidopsis*, variations in the H170 residue reduced the binding of AtARF5 to the corresponding AuxREs, as did mutations identified from the P218, R215, T227, and S230 codons. Interrupted dimerization was identified as results of G279, A282, and A287 substitution identified in the ARF domain. In our alignments of the 76 sequences, residues among those sites were carefully scanned and we found strong associations between conserved codon patterns and transcription expression patterns (**Supplementary Figure 1**), with substitution from H to G at the H170 residue position or substitution of T to A at the 202 residue position resulting in reduced expression of ARFs from group III. Since motifs and some amino acid residues from the ARF (MR) domain play a substantial role in binding target DNA, it might be plausible to postulate that down-regulation of expression of *ARF*s was associated with truncated conserved domains or even substitution of an amino acid residue in such domains.

### Diversified *PgrARF*s and Potential Candidate Genes for Involvement in Seed Coat Development

ARFs regulate numerous auxin-related processes at different plant developmental stages, as evident from gene expression patterns identified from previous studies (Xing et al., 2011; Zouine et al., 2014; Hu et al., 2015). In the current study, two pairs of highly expressed *ARF*s (*PgrARF1* and *PgrARF*2, and *PgrARF7* and *PgrARF19*) exhibited broad-spectrum expression in several different plant tissues, and shared a similar expression pattern to those of *EucARF1* and *EucARF2*, and *EucARF17* and *EucARF9* from eucalyptus (Yu et al., 2014). On the other hand, tissue-specific expression patterns were identified from the four corresponding *ARF*s in other species, including *Arabidopsis*, tomato, and citrus (Okushima et al., 2005; Zouine et al., 2014; Li et al., 2015). In situations where functional analysis of *ARF*s has been studied, functional mutant analysis in *Arabidopsis* provided invaluable resources for exploring *ARF* gene functions in other species. For orthologs from *Arabidopsis*, the loss-of-function double mutant revealed overlapping functions of *AtARF9* and *AtARF17*, which participate in the key step of lateral root formation and root development (Okushima et al., 2005). *ARF1* and *ARF2* regulate leaf senescence and floral organ abscission, while sharing partial functional redundancy (Ellis et al., 2005).

Interestingly, we found that the expression patterns of *PgrARF1* and *PgrARF2* were also very similar, as were those of *PgrARF7* and *PgrARF19*. The similarity of expression pattern might be related to functional redundancy as occurred in their respective orthologs from *Arabidopsis*. Combined with the roles of *ARF1* and *ARF2* in cell division and cellulose synthesis, specifically the role of mediating cell division in seed coat development of *AtARF2* (Schruff et al., 2006; Hughes et al., 2008), and the similar expression patterns from a number of tissues between two genes, we proposed that *PgrARF1* and *PgrARF2* are two structurally intact candidates that participate in cell division of seed coat during seed coat development.

## Data Availability Statement

Publicly available datasets were analyzed in this study. This data can be found here: https://genome.jgi.doe.gov.

## Author Contributions

GQ conceived the pomegranate ARFs project. L’Y conducted the data analysis of the project and wrote the manuscript. CL, JL, XQ, and BJ conducted the fruits sampling, experiments, and data analysis. GQ and RM revised the manuscript.

## Funding

This work was supported by the Special project on Science and Technology of Anhui Province, China (201903b06020017) and Natural Science Foundation of Anhui Province (1708085MC85).

## Conflict of Interest

The authors declare that the research was conducted in the absence of any commercial or financial relationships that could be construed as a potential conflict of interest.

## Supplementary Material

Supplementary Figure 1 Codon usage of conserved residues from the auxin response factor (ARF) domain. Multiple sequence alignment of 76 protein sequences was performed by MUSCLE. The genomic location of conserved residues is highlighted and marked with triangles.

Supplementary S1 Amino acid sequences of 76 ARFs identified from four plant species

Supplementary S2 Gene ID correspondence of 76 *ARF*s from four plant species

Supplementary S3 Summary of structure and distribution of *ARF*s

Supplementary S4 Multiple alignment of ARFs from four plants

Supplementary S5 Duplication type of *ARFs* from four plant species

## References

[B1] Bailey T. L. Boden M. Buske F. A. Frith M. Grant C. E. Clementi L. (2009). MEME SUITE: tools for motif discovery and searching. Nucleic Acids Res. 37 (suppl_2), W202–W208. 10.1093/nar/gkp335 19458158 PMC2703892

[B2] Blanc G. Wolfe K. H. (2004). Functional divergence of duplicated genes formed by polyploidy during Arabidopsis evolution. Plant Cell 16 (7), 1679–1691. 10.1105/tpc.021410 15208398 PMC514153

[B3] Boer D. R. Freire-Rios A. van den Berg W. A. Saaki T. Manfield I. W. Kepinski S. (2014). Structural basis for DNA binding specificity by the auxin-dependent ARF transcription factors. Cell 156 (3), 577–589. 10.1016/j.cell.2013.12.027 24485461

[B4] De Jong M. Wolters-Arts M. Schimmel B. C. Stultiens C. L. de Groot P. F. Powers S. J. (2015). Solanum lycopersicum AUXIN RESPONSE FACTOR 9 regulates cell division activity during early tomato fruit development. J. Exp. Bot. 66 (11), 3405–3416. 10.1093/jxb/erv152 25883382 PMC4449553

[B5] Edgar R. C. (2004). MUSCLE: multiple sequence alignment with high accuracy and high throughput. Nucleic Acids Res. 32 (5), 1792–1797. 10.1093/nar/gkh340 15034147 PMC390337

[B6] Ellis C. M. Nagpal P. Young J. C. Hagen G. Guilfoyle T. J. Reed J. W. (2005). *AUXIN RESPONSE FACTOR1* and *AUXIN RESPONSE FACTOR2* regulate senescence and floral organ abscission in Arabidopsis thaliana. Development 132 (20), 4563–4574. 10.1242/dev.02012 16176952

[B7] Fang X. Zhang Y. Zhang Y. Huang K. Yang W. Li X. (2019). *De novo* transcriptome assembly and identification of genes related to seed size in common buckwheat (Fagopyrum esculentum M.). Breed. Sci. 69 (3), 487–497. 10.1270/jsbbs.18194 31598082 PMC6776140

[B8] Figueiredo D. D. Batista R. A. Roszak P. J. Hennig L. Köhler C. (2016). Auxin production in the endosperm drives seed coat development in Arabidopsis. Elife 5, e20542. 10.7554/eLife.20542 27848912 PMC5135394

[B9] Finet C. Fourquin C. Vinauger M. Berne-Dedieu A. Chambrier P. Paindavoine S. (2010). Parallel structural evolution of auxin response factors in the angiosperms. Plant J. 63 (6), 952–959. 10.1111/j.1365-313X.2010.04292.x 20626651

[B10] Finet C. Berne-Dedieu A. Scutt C. P. Marletaz F. (2013). Evolution of the ARF gene family in land plants: old domains, new tricks. Mol. Biol. Evol. 30 (1), 45–56. 10.1093/molbev/mss220 22977118

[B11] Garcia D. Fitz Gerald J. N. Berger F. (2005). Maternal control of integument cell elongation and zygotic control of endosperm growth are coordinated to determine seed size in Arabidopsis. Plant Cell 17 (1), 52–60. 10.1105/tpc.104.027136 15598800 PMC544489

[B12] Goetz M. Vivian-Smith A. Johnson S. D. Koltunow A. M. (2006). *AUXIN RESPONSE FACTOR8* is a negative regulator of fruit initiation in Arabidopsis. Plant Cell 18 (8), 1873–1886. 10.1105/tpc.105.037192 16829592 PMC1533983

[B13] Guilfoyle T. J. Hagen G. (2012). Getting a grasp on domain III/IV responsible for Auxin Response Factor–IAA protein interactions. Plant Sci. 190, 82–88. 10.1016/j.plantsci.2012.04.003 22608522

[B14] Guilfoyle T. J. (2015). The PB1 domain in auxin response factor and Aux/IAA proteins: a versatile protein interaction module in the auxin response. Plant Cell 27 (1), 33–43. 10.1105/tpc.114.132753 25604444 PMC4330575

[B15] Hardtke C. S. Berleth T. (1998). The Arabidopsis gene MONOPTEROS encodes a transcription factor mediating embryo axis formation and vascular development. EMBO J. 17 (5), 1405–1411. 10.1093/emboj/17.5.1405 9482737 PMC1170488

[B16] Hu B. Jin J. Guo A.-Y. Zhang H. Luo J. Gao G. (2014). GSDS 2.0: an upgraded gene feature visualization server. Bioinformatics 31 (8), 1296–1297. 10.1093/bioinformatics/btu817 25504850 PMC4393523

[B17] Hu W. Zuo J. Hou X. Yan Y. Wei Y. Liu J. (2015). The auxin response factor gene family in banana: genome-wide identification and expression analyses during development, ripening, and abiotic stress. Front. Plant Sci. 6, 742. 10.3389/fpls.2015.00742 26442055 PMC4569978

[B18] Hughes R. Spielman M. Schruff M. C. Larson T. R. Graham I. A. Scott R. J. (2008). Yield assessment of integument-led seed growth following targeted repair of auxin response factor 2. Plant Biotechnol. J. 6 (8), 758–769. 10.1111/j.1467-7652.2008.00359.x 18643948

[B19] Jiao Y. Wickett N. J. Ayyampalayam S. Chanderbali A. S. Landherr L. Ralph P. E. (2011). Ancestral polyploidy in seed plants and angiosperms. Nature 473 (7345), 97–100. 10.1038/nature09916 21478875

[B20] Jiao Y. Leebens-Mack J. Ayyampalayam S. Bowers J. E. McKain M. R. McNeal J. (2012). A genome triplication associated with early diversification of the core eudicots. Genome Biol. 13 (1), R3. 10.1186/gb-2012-13-1-r3 22280555 PMC3334584

[B21] Johnson L. S. Eddy S. R. Portugaly E. (2010). Hidden Markov model speed heuristic and iterative HMM search procedure. BMC Bioinf. 11 (1), 431. 10.1186/1471-2105-11-431 PMC2931519 20718988

[B22] Keane T. M. Creevey C. J. Pentony M. M. Naughton T. J. Mclnerney J. O. (2006). Assessment of methods for amino acid matrix selection and their use on empirical data shows that ad hoc assumptions for choice of matrix are not justified. BMC Evolution. Biol. 6 (1), 29. 10.1186/1471-2148-6-29 PMC1435933 16563161

[B23] Kim N. D. Mehta R. Yu W. Neeman I. Livney T. Amichay A. (2002). Chemopreventive and adjuvant therapeutic potential of pomegranate (Punica granatum) for human breast cancer. Breast Cancer Res. Treat 71 (3), 203–217. 10.1023/A:1014405730585 12002340

[B24] Korasick D. A. Westfall C. S. Lee S. G. Nanao M. H. Dumas R. Hagen G. (2014). Molecular basis for AUXIN RESPONSE FACTOR protein interaction and the control of auxin response repression. Proc. Natl. Acad. Sci. U.S.A. 111 (14), 5427–5432. 10.1073/pnas.1400074111 24706860 PMC3986151

[B25] Krzywinski M. Schein J. Birol I. Connors J. Gascoyne R. Horsman D. (2009). Circos: an information aesthetic for comparative genomics. Genome Res. 19 (9), 1639–1645. 10.1101/gr.092759.109 19541911 PMC2752132

[B26] Le B. Nawaz M. A. Rehman H. M. Le T. Yang S. H. Golokhvast K. S. (2016). Genome-wide characterization and expression pattern of auxin response factor (ARF) gene family in soybean and common bean. Genes Genomics 38 (12), 1165–1178. 10.1007/s13258-016-0462-y

[B27] Letunic I. Bork P. (2006). Interactive Tree Of Life (iTOL): an online tool for phylogenetic tree display and annotation. Bioinformatics 23 (1), 127–128. 10.1093/bioinformatics/btl529 17050570

[B28] Li S.-B. OuYang W.-Z. Hou X.-J. Xie L.-L. Hu C.-G. Zhang J.-Z. (2015). Genome-wide identification, isolation and expression analysis of auxin response factor (ARF) gene family in sweet orange (*Citrus sinensis*). Front. Plant Sci. 6, 119. 10.3389/fpls.2015.00119 25870601 PMC4378189

[B29] Liu Z. Miao L. Huo R. Song X. Johnson C. Kong L. (2018). ARF2-ARF4 and ARF5 are Essential for Female and Male Gametophyte Development in Arabidopsis. Plant Cell Physiol. 59 (1), 179–189. 10.1093/pcp/pcx174 29145642

[B30] Nagpal P. Ellis C. M. Weber H. Ploense S. E. Barkawi L. S. Guilfoyle T. J. (2005). Auxin response factors *ARF6* and *ARF8* promote jasmonic acid production and flower maturation. Development 132 (18), 4107–4118. 10.1242/dev.01955 16107481

[B31] Nesi N. Debeaujon I. Jond C. Stewart A. J. Jenkins G. II Caboche M. (2002). The TRANSPARENT TESTA16 locus encodes the *ARABIDOPSIS* BSISTER MADS domain protein and is required for proper development and pigmentation of the seed coat. Plant Cell 14 (10), 2463–2479. 10.1105/tpc.004127 12368498 PMC151229

[B32] Okushima Y. Mitina I. Quach H. L. Theologis A. (2005). *AUXIN RESPONSE FACTOR 2 (ARF2)*: a pleiotropic developmental regulator. Plant J. 43 (1), 29–46. 10.1111/j.1365-313X.2005.02426.x 15960614

[B33] Piya S. Shrestha S. K. Binder B. Stewart C. N. Jr. Hewezi T. (2014). Protein-protein interaction and gene co-expression maps of ARFs and Aux/IAAs in Arabidopsis. Front. Plant Sci. 5, 744. 10.3389/fpls.2014.00744 25566309 PMC4274898

[B34] Qin G. Xu C. Ming R. Tang H. Guyot R. Kramer E. M. (2017). The pomegranate (Punica granatum L.) genome and the genomics of punicalagin biosynthesis. Plant J. 91 (6), 1108–1128. 10.1111/tpj.13625 28654223

[B35] Qin G. Liu C. Li J. Qi Y. Gao Z. Zhang X. (2020). Diversity of metabolite accumulation patterns in inner and outer seed coats of pomegranate: exploring their relationship with genetic mechanisms of seed coat development. Hortic. Res. 7:10. 10.1038/s41438-019-0233-4 31934341 PMC6946660

[B36] Rambaut A. (2007). FigTree, a graphical viewer of phylogenetic trees. Computer Program.

[B37] Schruff M. C. Spielman M. Tiwari S. Adams S. Fenby N. Scott R. J. (2006). The *AUXIN RESPONSE FACTOR 2* gene of Arabidopsis links auxin signalling, cell division, and the size of seeds and other organs. Development 133 (2), 251–261. 10.1242/dev.02194 16339187

[B38] Shen C. Yue R. Sun T. Zhang L. Xu L. Tie S. (2015). Genome-wide identification and expression analysis of auxin response factor gene family in Medicago truncatula. Front. Plant Sci. 6, 73. 10.3389/fpls.2015.00073 25759704 PMC4338661

[B39] Singh V. K. Jain M. (2015). Genome-wide survey and comprehensive expression profiling of Aux/IAA gene family in chickpea and soybean. Front. Plant Sci. 6, 918. 10.3389/fpls.2015.00918 26579165 PMC4621760

[B40] Sun X. Shantharaj D. Kang X. Ni M. (2010). Transcriptional and hormonal signaling control of Arabidopsis seed development. Curr. Opin. Plant Biol. 13 (5), 611–620. 10.1016/j.pbi.2010.08.009 20875768

[B41] Wang R. Estelle M. (2014). Diversity and specificity: auxin perception and signaling through the TIR1/AFB pathway. Curr. Opin. Plant Biol. 21, 51–58. 10.1016/j.pbi.2014.06.006 25032902 PMC4294414

[B42] Wang D. Pei K. Fu Y. Sun Z. Li S. Liu H. (2007). Genome-wide analysis of the auxin response factors (ARF) gene family in rice (*Oryza sativa*). Gene 394 (1-2), 13–24. 10.1016/j.gene.2007.01.006 17408882

[B43] Wang Y. Tang H. DeBarry J. D. Tan X. Li J. Wang X. (2012). MCScanX: a toolkit for detection and evolutionary analysis of gene synteny and collinearity. Nucleic Acids Res. 40 (7), e49–e49. 10.1093/nar/gkr1293 22217600 PMC3326336

[B44] Wen J. Guo P. Ke Y. Liu M. Li P. Wu Y. (2019). The auxin response factor gene family in allopolyploid Brassica napus. PloS One 14 (4), e0214885. 10.1371/journal.pone.0214885 30958842 PMC6453480

[B45] Wilmoth J. C. Wang S. Tiwari S. B. Joshi A. D. Hagen G. Guilfoyle T. J. (2005). *NPH4/ARF7* and *ARF19* promote leaf expansion and auxin-induced lateral root formation. Plant J. 43 (1), 118–130. 10.1111/j.1365-313X.2005.02432.x 15960621

[B46] Xing H. Pudake R. N. Guo G. Xing G. Hu Z. Zhang Y. (2011). Genome-wide identification and expression profiling of auxin response factor (ARF) gene family in maize. BMC Genomics 12 (1), 178. 10.1186/1471-2164-12-178 21473768 PMC3082248

[B47] Yu H. Soler M. Mila I. San Clemente H. Savelli B. Dunand C. (2014). Genome-wide characterization and expression profiling of the *AUXIN RESPONSE FACTOR* (*ARF*) gene family in Eucalyptus grandis. PloS One 9 (9), e108906. 10.1371/journal.pone.0108906 25269088 PMC4182523

[B48] Zhang X. Yan F. Tang Y. Yuan Y. Deng W. Li Z. (2015). Auxin response gene *SlARF3* plays multiple roles in tomato development and is involved in the formation of epidermal cells and trichomes. Plant Cell Physiol. 56 (11), 2110–2124. 10.1093/pcp/pcv136 26412778

[B49] Zhao X. Yuan Z. Feng L. Fang Y. (2015). Cloning and expression of anthocyanin biosynthetic genes in red and white pomegranate. J. Plant Res. 128 (4), 687–696. 10.1007/s10265-015-0717-8 25810223

[B50] Zouine M. Fu Y. Chateigner-Boutin A. L. Mila I. Frasse P. Wang H. (2014). Characterization of the tomato ARF gene family uncovers a multi-levels post-transcriptional regulation including alternative splicing. PloS One 9 (1), e84203. 10.1371/journal.pone.0084203 24427281 PMC3888382

