# Supplementary figures and images for: Identification of Candidate Auxin Response Factors Involved in Pomegranate Seed Coat Development
Source: Front Plant Sci. 2020 Sep 15;11:536530. doi: 10.3389/fpls.2020.536530 (PMC7522551; doi:10.3389/fpls.2020.536530)

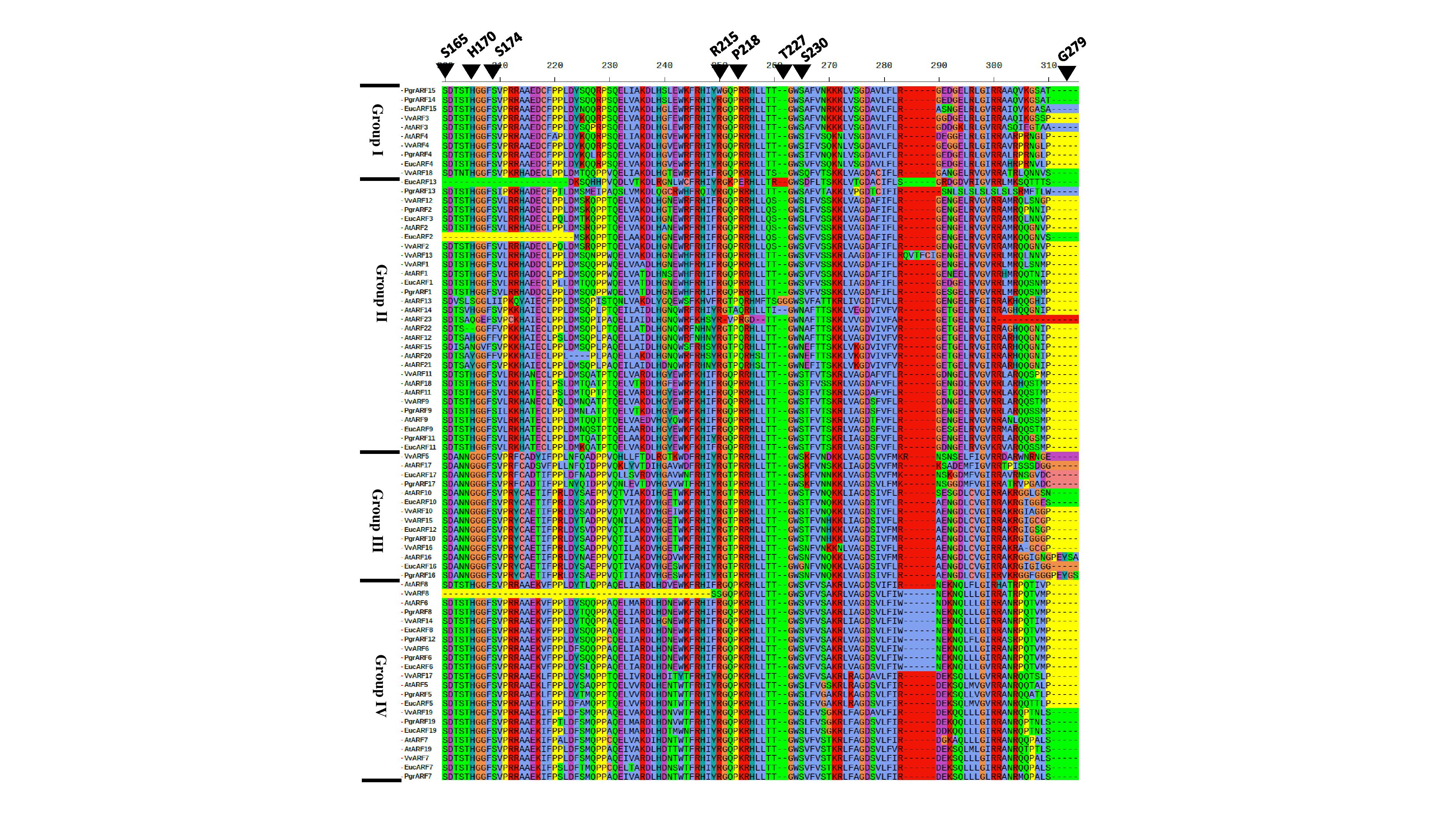

Supplement: Supplementary Figure 1 — Codon usage of conserved residues from the auxin response factor (ARF) domain. Multiple sequence alignment of 76 protein sequences was performed by MUSCLE. The genomic location of conserved residues is highlighted and marked with triangles. [file Image_1.jpeg]
